# Recent Advances on Boosting the Cell Voltage of Aqueous Supercapacitors

**DOI:** 10.1007/s40820-020-00430-4

**Published:** 2020-04-20

**Authors:** Qianzhi Gou, Shuang Zhao, Jiacheng Wang, Meng Li, Junmin Xue

**Affiliations:** 1 MOE Key Laboratory of Low-Grade Energy Utilization Technologies and Systems, CQU-NUS Renewable Energy Materials and Devices Joint Laboratory, School of Energy and Power Engineering, Chongqing University, Chongqing, 400044 People’s Republic of China; 2 Department of Materials Science and Engineering, CQU-NUS Renewable Energy Materials and Devices Joint Laboratory, National University of Singapore, Singapore, 117573 Singapore

**Keywords:** Aqueous supercapacitors, Cell voltage, Electrodes, Electrolytes, Asymmetric design

## Abstract

Due to its ultra-fast charge/discharge rate, long cyclic life span, and environmental benignity, aqueous supercapacitor (SC) is considered as a proper next-generation energy storage device. Unfortunately, limited by undesirable water electrolysis and unreasonable electrode potential range, aqueous SC normally generates a narrow cell voltage, resulting in a low energy density. To address such challenge, enormous efforts have been made to construct high-voltage aqueous SCs. Despite these achievements, the systematic reviews about this field are still rare. To fill this knowledge gap, this review summarizes the recent advances about boosting the cell voltage of aqueous SCs. From the viewpoint of electrode, doping alkali cations, modulating the electrode mass ratio, and optimizing the surface charge density are regarded as three effective pathways to achieve this goal. However, adjusting the appropriate pH level, introducing redox mediators, and constructing “water-in-salt” electrolyte are other three universal routes from the electrolyte aspect. Furthermore, it is also effective to obtain the high-voltage aqueous SCs through asymmetric design, such as designing asymmetric SCs. The confronting challenges and future development tendency towards the high-voltage aqueous SCs are further discussed.

## Highlights

- High-voltage aqueous supercapacitors hold promise for commercial energy storage devices due to the excellent electrochemical performance.
- This review summarizes the efficacious measures on boosting the cell voltage of aqueous supercapacitors from the aspects of electrode, electrolyte, and asymmetric design.

## Graphical abstract

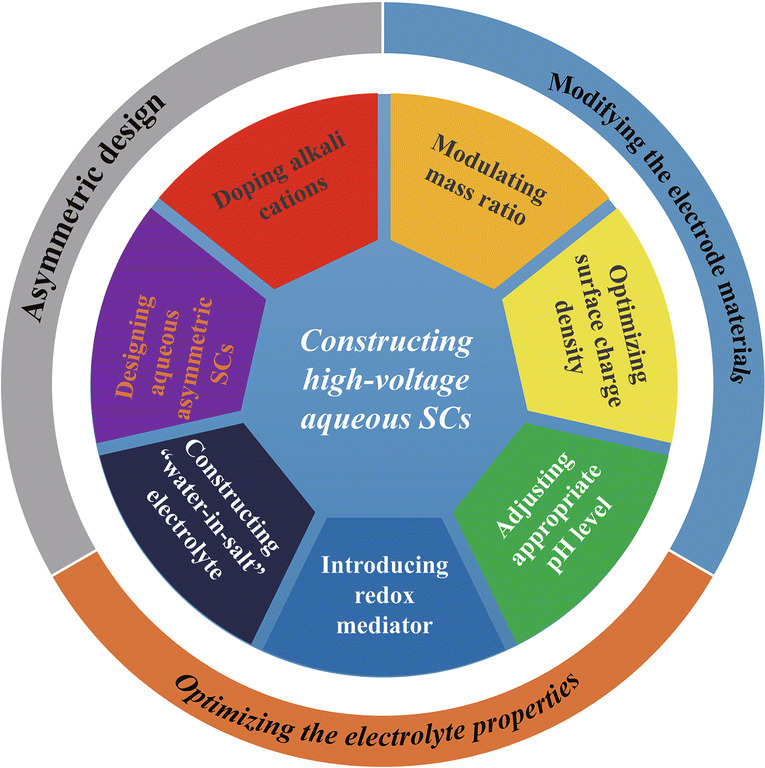

## Introduction

**Fig. 1:**
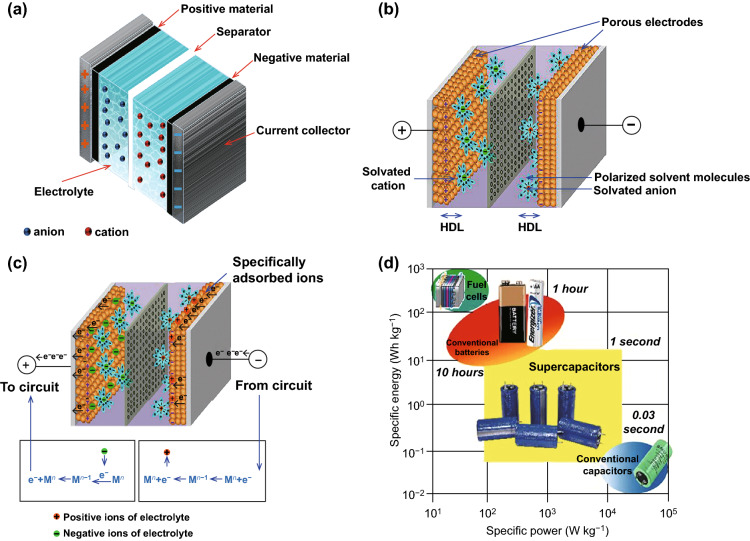
**a** Schematic of an intact SC device. **b** Charge storage mechanisms of EDLCs and PCs. **c** Charge storage of PCs. Adapted with permission from Ref. [10]. **d** Specific power versus specific energy plot for various types of energy storage devices. Adapted with permission from Ref. [12]

With the consumption of traditional fossil fuels, the ever-increasing energy depletion crisis and environmental pollution issues have drawn tremendous attentions in the past decades. To mitigate the aforesaid dilemma, it is urgent to develop numerous sustainable and renewable energy conversion and energy storage devices, such as secondary batteries [1, 2], supercapacitors (SCs) [3, 4], and fuel cells [5]. Among them, SCs, also named electrochemical capacitor, is recognized as an attractive candidate for next-generation energy storage systems, ascribed to its rapid charge/discharge rate, high power density, long cycling lifespan, favorable safety, and environmental benignity [6]. Similar to the majority of electrochemical energy storage devices, traditional commercial SCs are primarily composed of the positive electrode, negative electrode, electrolyte, current collector, and separator (Fig. 1a) [7, 8]. Based on the diverse energy storage mechanisms, SCs can be mainly classified into electrochemical double-layer capacitors (EDLCs) and pseudo-capacitors (PCs) (Fig. 1b, c) [7, 9, 10]. In principle, the charge storage mechanism of EDLCs is the electrostatic interaction at the interface between electrode and electrolyte, whereas PCs utilize the fast and reversible faradaic redox reactions to store charges [11]. Moreover, SCs can also be categorized into symmetric SCs (two identical electrodes) and ASCs (two dissimilar electrodes) according to the distinct structural configurations. Although great achievements have been obtained in pursuit of high performance SCs, the insufficient energy density is still hampering their practical applications (Fig. 1d) [12]. To satisfy the ever-increasing requirements of energy storage, it is desirable to establish the SC devices with high energy density.

According to the equation of energy density for SCs (*E* = 0.5*CV*^2^), the value of energy density (*E*) is directly determined by specific capacitance (*C*) and cell voltage (*V*). Up to now, enormous research hot spots mainly focus on how to design high specific capacitance electrode materials (e.g., carbon-based materials [4, 13], transition metal oxides [6, 14], conducting polymers [15, 16], and sulfides [17, 18] etc.), but neglect the other promising direction. In comparison with specific capacitance, energy density is proportional to the square of cell voltage (*V*^2^). Hence, it is more efficient to enhance the energy density of SCs via increasing the value of *V* [19, 20]. It is well known that the cell voltage is principally determined by the electrochemical stability window (ESW) of electrolyte [21]. Therefore, the major strategy to increase the cell voltage of aqueous SCs is selecting the electrolyte with a wide ESW [22]. At present, organic electrolyte is widely applied to assemble high-voltage SCs due to its high electrolysis resistance [23]. Nevertheless, the characteristics of inferior ionic conductivity, high flammability and volatility, toxicity as well as expensive price are the dominating shortcomings that restrict its practical prospects [24, 25]. Furthermore, although the SC device operating in organic electrolyte is able to deliver an enhanced energy density, the relatively high equivalent series resistance and low ion diffusion rate are still its fatal shortcomings which may sacrifice the additional power density (*P* = *V*^*2*^*/*4*R*_S_, where *P*, *V*, and *R*_S_ refer to power density, cell voltage, and equivalent inner resistance of the SC device, respectively) [26]. Therefore, it is desirable to search for more appropriate electrolytes to displace the organic electrolytes.

Among various candidates, aqueous electrolyte is re-considered as a proper substitute for organic electrolyte owing to its intriguing features, such as superior conductivity, low cost, high mobility, prominent safety, nontoxicity, and environmental benignity [9, 27]. The relevant works about aqueous SCs have received substantial attentions in recent years. Nevertheless, the practical applications of aqueous SCs are still inhibited by two major bottlenecks. One is the narrow ESW of aqueous media. As we all know, the thermodynamic stable potential range of H_2_O is only 1.23 V. When the applied potential is out of this value, H_2_ will be released on the surface of negative electrode resulting from the occurrence of hydrogen evolution reaction (HER), whereas O_2_ will be released on the positive electrode surface originating from oxygen evolution reaction (OER) process [21]. Along with these reactions, the actual ESW of aqueous media is normally lower than 1.23 V which may reduce the cell voltage of integrated device. Aiming at the above phenomenon, improving the over-potential of HER and OER under the steady state of electrode can efficiently solve this problem [26]. The other is the unsuitable electrode potential range. Owing to the different storage energy mechanisms, positive electrode and negative electrode possess their separate potential range [28]. When they are assembled as an intact aqueous SCs, the cell voltage is dictated by two electrodes. Unfortunately, limited by the unused potential region and electrocatalytic activity of electrodes in aqueous media, the cell voltage of integrated device is unequal to the sum of the theoretical potential ranges of both electrodes [29, 30]. Based on the aforesaid considerations, a great deal of efforts has been made to overcome these challenges, such as optimizing the features of electrodes, extending the ESW of electrolyte, and designing aqueous ASCs [20, 25, 31]. Despite these great achievements, the number of systematic reviews about boosting the cell voltage of aqueous SCs is still rare in recent years. Hence, the purpose of this mini review is to fill this knowledge gap.

Through further surveying and summarizing the domestic and overseas research works, it can be concluded that modifying the electrode materials, optimizing the electrolyte properties, and  designing asymmetric devices are regarded as three dominant and efficient strategies to acquire the high-voltage aqueous SCs. On this basis, this mini review is mainly divided into three sections and further expanded by detailed: (1) doping alkali cations, modulating the electrode mass ratio, and optimizing the surface charge density are three generic pathways from the aspect of electrodes; (2) adjusting the appropriate pH level, introducing redox mediators, and constructing “water-in-salt” (WIS) electrolyte are three universal methods from the viewpoint of electrolyte; (3) designing aqueous ASCs is an attractive pathway to widen the cell voltage from the device configuration aspect. The confronting challenges and development trends towards high-voltage aqueous SCs will be further discussed and forecasted.

## Modifying the Electrode Materials

The narrow cell voltage is a major obstacle that inhibits the development of aqueous SCs with high energy density. Considering the importance of electrodes, it is a feasible pathway to enlarge the cell voltage via modifying the electrodes materials [29, 32, 33]. Moreover, based on the various energy storage mechanisms, electrode materials can mainly be grouped into electric double-layer capacitive electrodes and pseudo-capacitive electrodes [34]. Among them, the former stores energy through the electrostatic interaction between the electrode and electrolyte, such as carbon-based materials, whereas the latter utilizes the fast and reversible redox reactions to store pseudo-capacitances, such as transition-metal compounds and conducting polymers [35, 36]. Up to now, tremendous attempts have been made to assemble the high-voltage aqueous SCs via modifying these two type electrodes, such as doping alkali cations, modulating the electrode mass ratio, and optimizing the surface charge density. On this basis, the relevant mechanisms and research status about aforesaid approaches are introduced and discussed in the following sections.

### Doping Alkali Cations

**Fig. 2:**
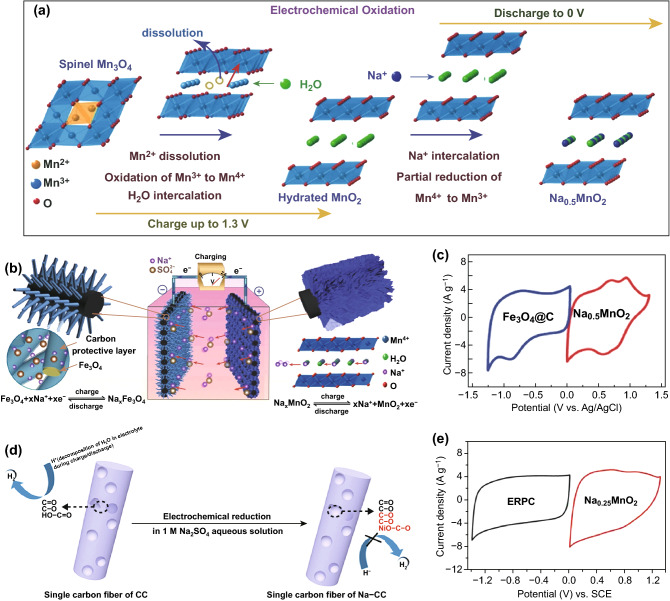
**a** Structural evolution process of Na_0.5_MnO_2_ during the electrochemical oxidation. **b** Electrode designs and charge-storage mechanism of the Fe_3_O_4_@C anode and the Na_0.5_MnO_2_ NWAs cathode in the aqueous ASC system. **c** CV curves of the Na_0.5_MnO_2_ NWAs and Fe_3_O_4_@C electrodes in separate potential ranges at a scan rate of 10 mV s^−1^. Adapted with permission from Ref. [31]. **d** Reaction schematic of alkali cations (Na^+^) on carbon-based electrodes. Adapted with permission from Ref. [28]. **e** CV curves of the Na_0.25_MnO_2_ and the ERPC electrodes in separate potential ranges at a scan rate of 25 mV s^−1^. Adapted with permission from Ref. [43]

Recently, doping alkali cations (Li^+^, Na^+^, and K^+^) into electrode materials is recognized as an efficacious strategy to extend the cell voltage of aqueous SCs [22, 31]. In principle, alkali cations exhibit various reaction mechanisms in different type electrode materials. Taking manganese oxide (MnO_2_) as a typical case, due to its charming properties of high specific capacitance, luxuriant reserves, environmental friendliness, low OER activity, and well-established fabrication methods, MnO_2_ is regarded as an ideal pseudo-capacitive electrode for aqueous SCs [37, 38]. When alkali cations are inserted into MnO_2_ framework, they can stabilize the polymorph structure, enhance cyclic stability, offer extra capacitance, and facilitate the ion diffusion process [39–42]. More importantly, alkali cations can also efficiently inhibit the electrocatalytic activity of MnO_2_ electrode in aqueous electrolyte because of the competitive relationship between alkali cations intercalation process and OER [31, 43]. For example, Jabeen et al. fabricated a hierarchical architecture of Birnessite Na_0.5_MnO_2_ nanosheet assembled nanowall arrays (NWAs) in situ grown on carbon cloth via an electrochemical oxidation method (Fig. 2a) [31]. Benefiting from the existence of Na^+^, the potential range of as-prepared Na_0.5_MnO_2_ NWAs electrode can be enlarged to 0–1.3 V (vs. Ag/AgCl) in a three-electrode system. To complement this electrode, carbon-coated Fe_3_O_4_ (Fe_3_O_4_@C) nanorods arrays were synthesized as anode and presented a stable potential range of 1.3–0 V (vs. Ag/AgCl). Benefiting from the smart electrode modification and unique fabrication methods, this Na_0.5_MnO_2_//Fe_3_O_4_@C aqueous ASC with 1 M Na_2_SO_4_ as the electrolyte can demonstrate a maximum cell voltage of 2.6 V (Fig. 2b, c).

Similarly, doping alkali cations is also suitable for extending the potential range of electric double-layer capacitive electrodes. As a representative for such electrodes, carbon-based materials have a long studying history in the area of aqueous SCs due to their favorable conductivity, superior chemical stability, inferior HER activity, and low cost [13, 44]. In general, carbonaceous electrodes normally possess the features of high specific surface area and porous structure, thus inevitably resulting in a mass of residual heteroatoms (N and O) on their surface which originated from the high temperature pyrolysis of organics and biomass in insert atmosphere [28, 45]. However, the existence of such heteroatoms is a double-edged sword. On the one hand, these heteroatoms can enhance electrical conductivity, offer abundant active sites, and accelerate the rate of electrochemical reactions. On the other hand, they can also improve the activity of HER and promote water electrolysis process. In view of this, doping alkali cations into carbon-based electrode can validly eliminate this scruple. When alkali cations are adsorbed on the defect positions of heteroatoms, they can not only keep the intrinsic advantages of heteroatoms, but also serve as physical barrier for H^+^ adsorption via an electrostatic repulsion behavior, thereby suppressing the activity of HER (Fig. 2d) [46, 47]. Recently, Xiong et al. employed an electroreduction method to fabricate electrochemically reduced porous carbon (ERPC) electrode in 1 M Na_2_SO_4_ solution [43]. It is notable that the electrochemical reduction technique enables Na^+^ to be adsorbed on the defect atom sites of the carbon-based materials through a thermodynamically favorable process. Owing to the physical barrier behavior of Na^+^, the potential range of this ERPC electrode was expanded to 1.4–0 V (vs. saturated calomel electrode, SCE). To simultaneously boost the potential range of positive electrode, they also inserted Na^+^ intoMnO_2_ electrode (Na_0.25_MnO_2_) via a facile electrochemical oxidation technique, which delivered a wide potential range of 0–1.3 V (vs. SCE). When both electrodes were assembled as a standard Na_0.25_MnO_2_//ERPC aqueous ASC device, this system was able to display a high cell voltage of 2.7 V (Fig. 2e). Motivated by the above studies, Wu et al. fabricated a 2.7 V aqueous ASC device which applied the Na-adsorbed graphene aerogels thin slice on the carbon cloth substrate (Na-FG-CC) as the negative electrode and the Na-adsorbed carbon layer coated Mn_3_O_4_ (C@Mn_3_O_4_) as the positive electrode, separately [48]. Moreover, Qin et al. also assembled a 2.1 V aqueous carbon-based SC which is composed of heteroatom-rich micropore carbon fiber fabric (CC) as cathode and Na-containing functional groups CC (Na-CC) as anode, respectively [28].

So far, Na^+^ is reported as the most common alkali cation to enlarge the electrode potential range. In aqueous media, Na^+^ will be hydrated by surrounding water molecules to form hydrated Na^+^ particles. Due to the strong Na^+^–H_2_O interaction, the radius of hydrated Na^+^ can even attain to 3.58 Å, resulting in the inferior mobility and conductivity. Consequently, it is necessary to search for other hydrated alkali cations with the smaller hydration radius. Among various candidates, the radius of hydrated K^+^ is only 3.31 Å and this property endows the hydrated K^+^ possesses the superior conductivity and mobility during the charge/discharge process. Recently, Xiong et al. assembled a 2.4 V aqueous SC device comprised of K_0.6_MnO_2_ (positive electrode), K^+^-adsorbed holey carbon (negative electrode), and 1 M KTFSI solution (electrolyte) [39]. Benefiting from the K^+^ modification, this K_0.6_MnO_2_//KHC device could deliver a 52.8 Wh kg^−1^ at 500 kW kg^−1^ and 95% capacitance retention after 10,000 cycles at 20 Ag^−1^. In addition, to further contrast the applications of doping alkali cations in the field of high-voltage aqueous SCs, these aforesaid recent works are also exhibited in Table 1.

**Table 1:** The comparison of recent works about doping alkali cations in the field of high-voltage aqueous SCs

Electrode materials	Electrolyte	Alkali cations	Cell voltage (V)	Energy density (Wh kg^−1^)/power density (W kg^−1^)	References
Na_0.5_MnO_2_( +)//Fe_3_O_4_@C( −)	1 M Na_2_SO_4_	Na^+^	2.6	81/647	[31]
Na_0.25_MnO_2_( +)//ERPC( −)	1 M Na_2_SO_4_	Na^+^	2.7	61.1/982	[43]
C@Mn_3_O_4_( +)//Na-FG-CC( −)	1 M Na_2_SO_4_	Na^+^	2.7	110.4/1352	[48]
CC( +)//Na-CC( −)	1 M Na_2_SO_4_	Na^+^	2.1	–	[28]
K_0.6_MnO_2_( +)//KHC( −)	1 M KTFSI	K^+^	2.4	52.8/500	[39]

As mentioned above, doping alkali cations into electrode materials is an effective and universal method to broaden the cell voltage of aqueous SCs. In general, this strategy can not only modify the electric double-layer capacitive electrodes, but also optimize the pseudo-capacitive electrodes. In other words, this strategy is suitable for boosting the cell voltage of aqueous symmetric SCs and ASCs. Additionally, selecting appropriate electrodes is a crucial factor for the success of this strategy. Normally, the host materials should possess porous structure which can provide abundant channels for cations intercalation and reactive sites for cations adsorption process. Besides the alkali cations listed above, more doping particles with high mobility and conductivity should be explored in the future works.

### Modulating the Electrode Mass Ratio

Except for the electrocatalytic activity of electrodes, the unsuitable electrode potential range is another adverse factor to impede the development prospects of high-voltage aqueous SCs. As reported by the previous studies, the upper limit potential (*P*_U_) and low limit potential (*P*_L_) are two vital parameters to determine the maximum cell voltage of aqueous SCs [49, 50]. Theoretically, the maximum cell voltage of aqueous SCs should be equal to the difference between *P*_U_ and *P*_L_ (*P*_U_–*P*_L_). Nevertheless, the practical cell voltage is very difficult to reach the theoretical value due to the restriction of potential of zero voltage (*P*_0V_). In aqueous SC devices, *P*_0V_ represents the cutoff potential of both cathode and anode when the device operates at 0 V [49]. Generally, the potential range of negative and positive electrode refers to *P*_L_ to *P*_0V_ and *P*_0V_ to *P*_U_, respectively. Limited by the intrinsic features of electrodes, the location of *P*_0V_ normally deviates from the middle value of (*P*_U_–*P*_L_) and thus leads to the potential range of cathode which is unequal to the anode [51]. In such a condition, one electrode will reach the limit potential (*P*_U_ or *P*_L_) sooner than the other electrode and the voltage region of the latter will not be fully exploited during the charge/discharge process [49, 52]. To address this challenge, it is worthwhile searching for efficacious measures to optimize the electrode potential range. As an attractive method, modulating the electrode mass ratio has received significant attentions in recent years and the mechanism of this strategy is displayed as Eqs. 1 and 2 [53]:

1 $$Q = C \times m \times \Delta E$$

2 $$\frac{{m_{ + } }}{{m_{ - } }} = \frac{{C_{ - } \times \Delta E_{ - } }}{{C_{ + } \times \Delta E_{ + } }}$$

**Fig. 3:**
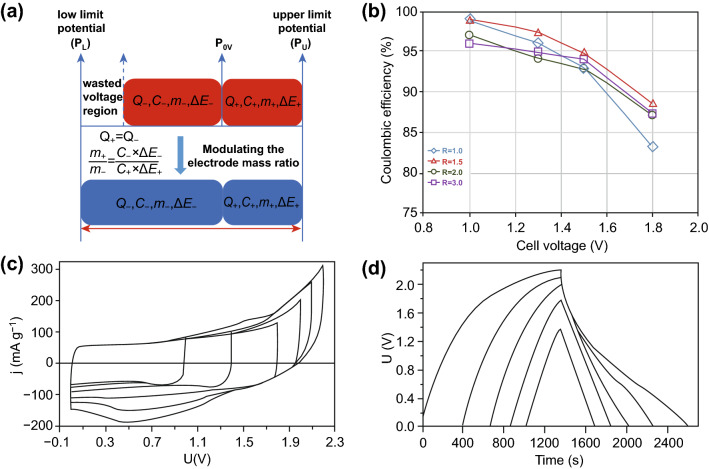
**a** Mechanism illustration showing the strategy to increase the cell voltage of aqueous SCs through modulating the electrode mass ratio. **b** Function relationship between coulombic efficiency and operating voltage for the carbon xerogel-based asymmetric systems with different electrode mass ratios. Adapted with permission from Ref. [55]. **c** CV curves of MnO_2_//AC device operating in 0.5 M Na_2_SO_4_ with mass ratios of 2.5 at scan rate of 2 mV s^−1^. **d** Galvanostatic charge/discharge curves of MnO_2_//AC device for mass ratio of 2.5. Adapted with permission from Ref. [19]

In Eq. 1, the parameters of *Q*, *C*, *m*, and Δ*E* stand for the surface charge stored in each electrode, specific capacitance, active mass, and potential range, respectively. Normally, the stored charges of positive electrode should be equal to the negative electrode (*Q*_+_ = *Q*_−_). Otherwise, the as-assembled device is unable to guarantee to work reversibly during the repeated cycling process [50]. In Eq. 2, the parameters of *m*_+_ and *m*_−_ refer to the active mass of two electrodes. Whereas *C*_−_ and Δ*E*_−_ are the specific capacitance and potential window of anode, *C*_+_ and Δ*E*_+_ represent the specific capacitance and potential window of cathode. According to the two equations, we can conclude that the electrode mass ratio is a vital factor for determining the electrode potential range (Fig. 3a). To acquire the high-voltage aqueous SCs, one of the promising strategies is to modulate the electrode mass ratios [30, 54].

For example, Calvo et al. designed aqueous ASCs consisted of carbon xerogel electrodes with various mass ratios. Based on the experimental results, this assembled device could yield a maximum cell voltage of 1.8 V when the optimum electrode mass ratio was close to around 2 (Fig. 3b) [55]. Similarly, Demarconnay et al. assembled a high-voltage MnO_2_//activated carbon (AC) aqueous ASC device through optimizing the electrode mass ratio [19]. When an integrated system was composed of both electrodes with the optimal mass ratio (2.5), the maximum cell voltage of this device could attain to 2 V (Fig. 3c, d). Besides, Wang et al. also utilized this strategy to explore the relationship between the electrode mass ratio and the cell voltage of aqueous SCs consisted of polyaniline (PANI) and RuO_2_ [56]. For the PANI//PANI and RuO_2_//RuO_2_ systems, these devices could achieve the maximum cell voltage when the electrodes mass ratio was on the verge of around 1, whereas the RuO_2_//PANI device with an electrode mass ratio of 0.81 will yield the maximum cell voltage in comparison with the SC system with an electrode mass ratio of 0.26 and 1.09.

In summary, modulating the electrode mass ratio is a satisfactory approach possessing the advantages of simple operation and low cost to enlarge the cell voltage of aqueous SCs, which seems to be suitable for large-scale industrial applications. According to Eqs. 1 and 2, it is convenient to calculate the theoretical optimal electrode mass ratio. After the precise adjustment, the utilization rate of electrode potential range could be significantly expanded. Nevertheless, this strategy cannot adjust the location of *P*_0V_, leading to the improvement of energy density is insufficient. In addition, due to the lack of scientific analysis methods, it is complicated and time-consuming to acquire the practical optimal electrode mass ratio through numerous experimental gradient designs.

### Optimizing the Surface Charge Density

**Fig. 4:**
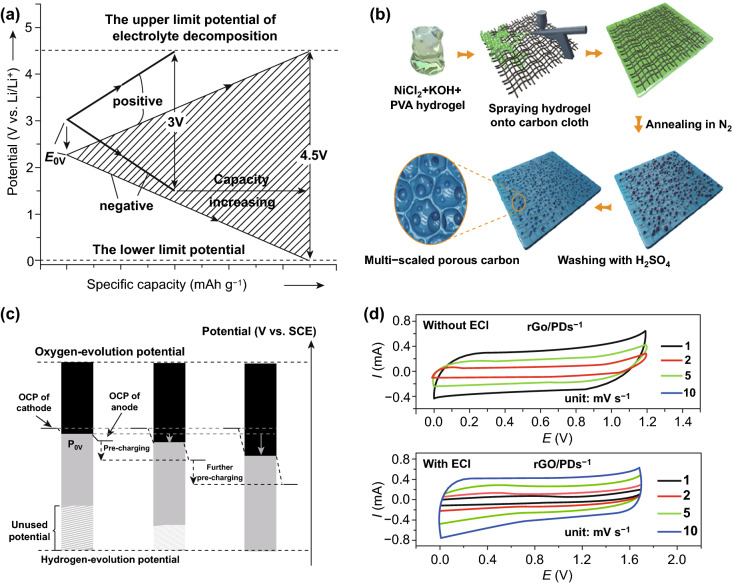
**a** Mechanism illustration of optimizing the surface charge density to extend the electrode potential window. The shadowed area is equal to the value of the energy density. Adapted with permission from Ref. [51]. The illustration of **b** the synthesis procedure of MSPC on carbon cloth and **c** tuning the electrode potential range downward through pre-charging treatment. Adapted with permission from Ref. [59]. **d** CV curves of rGO/PDs-1-based FSC without (above) and with ECI (below). Adapted with permission from Ref. [60]

In addition to the electrode mass ratio, the surface charge density is another pivotal factor to affect the electrode potential range [51]. Hence, it is feasible to enlarge the electrode potential range through optimizing the surface charge density. Compared with modulating the electrode mass ratio, this strategy is convenient and high efficiency to gain the appropriate electrodes. Besides, it can simultaneously increase the potential range and capacitance of electrodes through adjusting the location of *P*_0V_ (Fig. 4a).

According to the previous reports, electrochemical charge injection (ECI) technique is normally applied to induce the variations of charge density via changing the initial potential of electrodes [26, 57]. Based on the various reaction mechanisms, ECI technique is mainly divided into two generic methods: Faradaic and non-Faradaic charge injection. In Faradic charge inject, it employs the Faradic reactions with counter ions to induce the variations of electrode charge density in electrolytes. While, the non-Faradic charge injection relies on the double-layer adsorption of counter ions to alter the charge density [51]. Due to its high reversibility, the non-Faradic charge injection is more suitable for the most energy storage devices. On this basis, the applications of ECI technique have received substantial attentions in recent years. For instance, the ECI technique was applied for a V_2_O_5_//AC device operating in 2 M LiNO_3_ electrolyte [58]. When the initial potential of both electrodes was adjusted to  0.2 V (vs. SCE), this device could afford a maximum cell voltage of 1.8 V.

Similarly, this technique also holds great promise to extend the cell voltage of aqueous symmetric SCs. Recently, Yu et al. applied the ECI technique to boost the cell voltage of a multi-scale porous carbon (MSPC)-based aqueous symmetric SC [59]. Through elaborately selecting the pre-charging potential and tuning the *P*_0V_ downward, the maximum cell voltage was significantly expanded from 1.4 to 1.8 V (Fig. 4b, c). Subsequently, Guo et al. also widened the cell voltage of reduced graphene oxide/polymer dots (rGO/PDs)-based flexible symmetric SC (FSC) via the ECI technique [60]. After the adjustment of surface charge density, this built device could produce a maximum cell voltage of 1.7 V, much higher than that of 1.2 V for FSC without CEI treatment (Fig. 4d).

From the examples above, it is a rational and high-efficiency strategy to expand the cell voltage of aqueous SCs via optimizing the surface charge density. As an available method, ECI technique can validly tune the surface charge density through adjusting the position of *P*_0V_. However, this strategy cannot accurately control the degree of pre-charging treatment due to the self-discharge behavior which may reduce the energy utilization efficiency of aqueous SCs [49, 59]. Hence, how to mitigate the adverse impact of self-discharge phenomenon is the pivotal challenge for this strategy. Moreover, apart from CEI technique, more effective techniques should be explored to optimize the surface charge density, such as applying thermo-electrochemical effects and introducing different functional groups [26, 44, 61].

## Optimizing the Electrolyte Properties

Besides electrode materials, electrolyte is another pivotal component for aqueous SCs [53]. Due to the restriction of undesirable water electrolysis, aqueous electrolyte normally generates a narrow ESW and thus hampers the application prospect of aqueous SCs. As for this bottleneck, adjusting an appropriate pH level, introducing redox mediators, and constructing “water-in-salt” electrolyte are regarded as three popular and efficient pathways to boost the cell voltage of aqueous SCs in recent years [62–64]. In view of this, the relevant mechanisms and research advances of these approaches will be introduced in the subsequent sections.

### Adjusting the Appropriate pH Level

Generally, the PH level in electrolyte represents the concentration of H^+^ and OH^−^. Aqueous electrolytes can be mainly classified into acidic (e.g., H_2_SO_4_), alkaline (e.g., KOH), and neutral (e.g., Na_2_SO_4_) based on their pH value [7, 53]. More recently, researchers have found that some electrochemical reactions in aqueous media are greatly affected by the pH level  of electrolyte, such as faradic redox reactions and water electrolysis process (e.g., HER and OER) [26]. To investigate the relationship between electrochemical reactions and electrolyte pH levels, two half reactions of water decomposition in neutral medium and corresponding Nernst equations are displayed as Eqs. 3 and 4 [65]:

3 $$[ + ]\quad 2{\text{H}}_{2} {\text{O}} \Leftrightarrow {\text{O}}_{2} + 4{\text{e}}^{ - } + 4{\text{H}}^{ + } \quad {\text{E}}_{{{\text{ox}}}} = 1.23 - 0.059\,{\text{pH}}$$

4 $$[ - ]\quad {\text{H}}_{2} {\text{O}} + 2{\text{e}}^{ - } \Leftrightarrow {\text{H}}_{2} + 2{\text{OH}} - {\text{E}}_{{{\text{red}}}} = - 0.059\,{\text{pH}}$$

In view of Eqs. 3 and 4, it can be deduced that pH level is strongly correlated with the water electrolysis activity. According to the Pourbaix diagram, the maximum ESW of aqueous electrolyte will be obtained when the oxidation reaction occurs in an acidic environment (low pH value), and the reduction reaction takes place in an alkaline condition (high pH value) [65, 66].

**Fig. 5:**
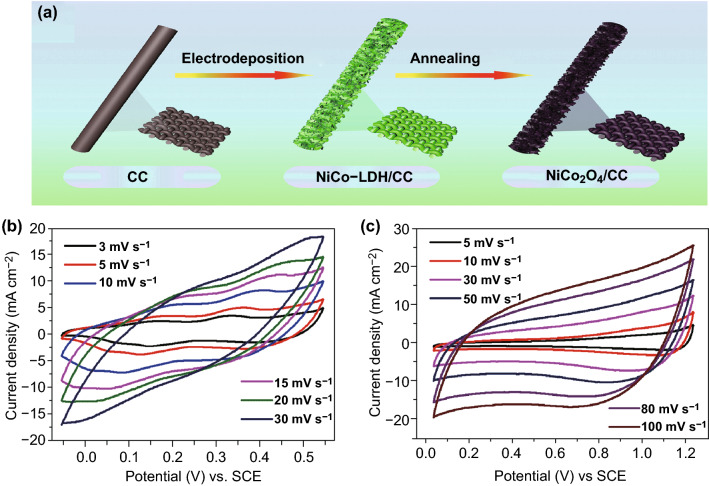
**a** Schematic illustration of the fabrication process of the NiCo_2_O_4_/CC composite. The CV profiles of the as-fabricated NiCo_2_O_4_/CC electrode at a three-electrode system **b** in alkaline electrolyte and **c** in neutral electrolyte with different scan rates. Adapted with permission from Ref. [68]

As mentioned before, adjusting an appropriate pH level of electrolyte is beneficial for improving the cell voltage of aqueous SCs. For instance, Khomenko et al. established a MnO_2_//AC device operating in various pH values electrolytes to verify the relationship between the over-potential of HER/OER and the pH values  of electrolyte [67]. According to the experimental results, the system would deliver a maximum cell voltage of 2 V when the electrolyte was 2 M KNO_3_ (pH = 6.4). Moreover, Zhao et al. contrasted the potential window of the as-prepared NiCo_2_O_4_/CC electrode in KOH alkaline electrolyte and mixed LiSO_4_/CoSO_4_ neutral electrolyte, respectively (Fig. 5a) [68]. In a three-electrode system, the potential range of NiCo_2_O_4_/CC electrode in mixed neutral electrolyte could be enlarged to 0–1.2 V (vs. SCE), which is superior to that measured in alkaline electrolyte (0–0.5 V) (Fig. 5b, c).

It is evident that these aforesaid cases merely verify that the pH level of electrolyte is greatly correlated with the cell voltage and ignore to take efficacious measures to adjust the pH value of electrolytes. In view of this, Slesinski et al. proposed a novel intrinsic pH gradient strategy to extend the cell voltage of neutral aqueous SCs (pH of both electrode/electrolyte interfaces differs by six units) [65]. Considering their superior donor–acceptor characters, the optimized carbon materials were applied as electrode materials. Through a modification with ammonia, the self-controlled pH gradient was introduced into this SC device and formed a protective layer on the electrode surface. In aqueous media, ammonia molecules not only increase the local pH, but also serve as protectors against electrode oxidation or electrolyte decomposition, thereby enlarging the ESW of aqueous electrolyte. Benefiting from this novel design, the as-synthesized aqueous SC system could exhibit a maximum cell voltage of 1.8 V. In addition, it is feasible to adjust the pH level of electrolytes via constructing the unique SC device. Recently, Wang et al. introduced a bipolar assembly of ion-exchange membranes (IEMs) as the separator for aqueous SC, which enabled cathode and anode to work in acidic and alkaline electrolyte, respectively [69]. Since the HER and OER process was blocked by the migration of H^+^ and OH^−^ confined in the acidic and alkaline electrolyte chambers, this built carbon-based SC device with dual aqueous electrolytes could deliver a wide cell voltage of around 1.8 V.

In conclusion, the ESW of aqueous media is greatly correlated with its pH value. Generally, the cell voltage of aqueous SCs with neutral electrolyte is wider than that of acidic/alkaline electrolyte owing to the low HER/OER activity. Hence, engineering an applicable electrolyte pH level is regarded as an effective approach to construct the high-voltage aqueous SCs. However, the above-mentioned modification measures are unable to fulfill the demands of industrial manufacture due to the complicated procedures and expensive price. As a result, it is urgent to search for more convenient and practical methods to adjust the appropriate pH value of electrolytes.

### Introducing Redox Mediators

**Fig. 6:**
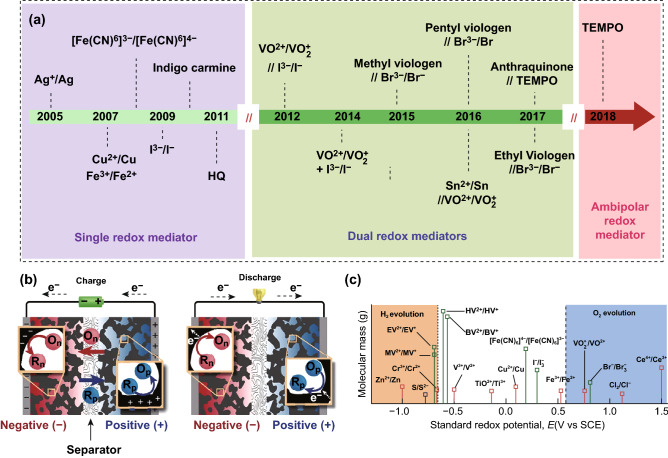
**a** Timeline of the development of redox couples in the field of SCs. HQ and TEMPO refer to hydroquinone and 2,2,6,6-tetramethylpiperidinyloxyl. Adapted with permission from Ref. [64]. **b** Schematic showing capacitive and faradic charge-storage processes of redox couples in electrolyte. **c** Reduction potentials of the couples considered relative to the thermodynamic stability window of water at neutral pH (white region). The lines colored in red, green, and blue are for couple stable in acidic, neutral, and basic conditions, respectively. BV: benzyl viologen, EV: benzyl viologen, HV: heptyl viologen, MV: methyl viologen, SCE: standard calomel electrode. Adapted from with permission Ref. [76]. (Color figure online)

Benefiting from the vast development prospect, constructing redox electrolyte has received various attentions to promote the performance of aqueous SCs in the past years (Fig. 6a) [64, 70, 71]. According to the various reaction mechanisms, redox electrolytes are mainly classified into redox-additives electrolytes (redox mediators are added into aqueous media to improve the electron-transfer kinetics on the electrode–electrolyte interface) and redox-active electrolytes (redox mediators are directly applied as aqueous electrolytes to undergo fast electron transfer reactions) [72, 73]. It should be pointed that redox mediator is the key component of redox electrolytes. Normally, redox mediators are divided into inorganic and organic redox mediators, respectively. The inorganic mediators (e.g., transition metal ions, halide ions, and cyanide ions) are the common redox additives for aqueous electrolyte, offering pseudo-capacitance via chemical valence variation, while the organic redox mediators (e.g., methylene blue, indigo carmine, and *p*-phenylenediamine) can achieve capacitance improvement via the conjugated effect [26, 64, 74]. Moreover, redox mediators can also be grouped into single and dual redox mediator based on the diverse species. When redox mediators are dissolved in aqueous solvent, the dissociative cations or anions will be adsorbed on the electrode surface through either electrostatic or physical behaviors and induce the extra redox reactions to increase the charge storage capacity [29, 75]. Consequently, the assembled aqueous SC operating in the redox electrolyte will substantially increase the energy density without sacrificing the power density (Fig. 6b) [75].

Besides the improvement of capacitance, this strategy is also suitable for increasing the cell voltage of aqueous SCs. It is well known that every redox mediator possesses a certain redox potential (Fig. 6c). During the charging process, the redox electrolyte will be oxidized (cathode) and reduced (anode) at the electrode surface, respectively. However, the variation of redox electrolyte is contrary during the discharge process. Along with the redox reaction, every redox mediator will exhibit an individual redox potential. If we select an appropriate redox mediator with redox potential near the H_2_O decomposition window, the cell voltage will be effectively boosted. This fact is attributed to the kinetic of redox mediator that is faster than water electrolysis, thus reducing the activity of water electrolysis [26, 76].

**Fig. 7:**
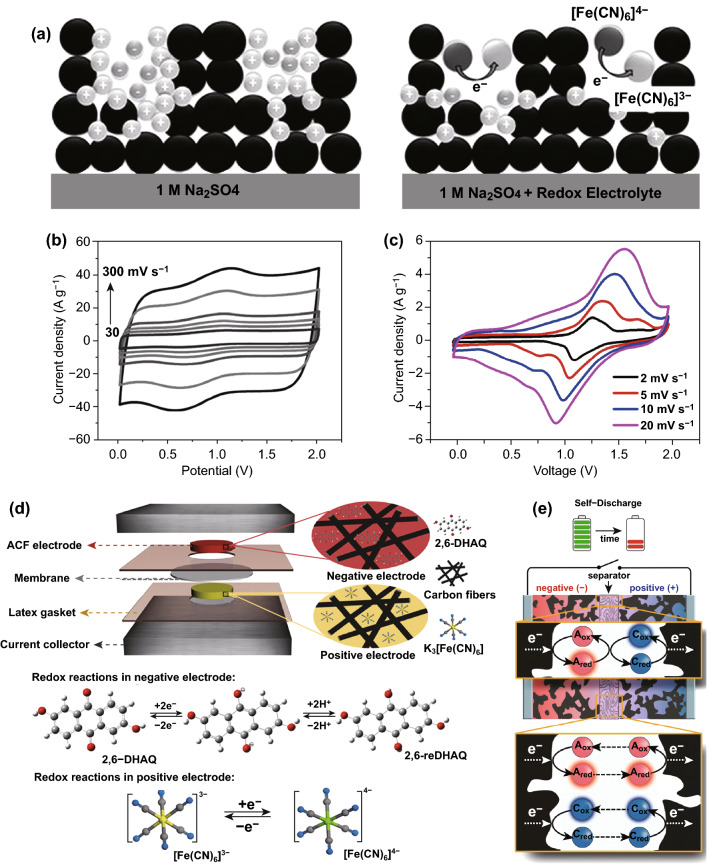
**a** Illustration of the charge storage mechanism in an carbon-based electrode using a 1.0 M Na_2_SO_4_ electrolyte (left) in the absence and (right) in the presence of a redox mediator. **b** CV curves of as-prepared carbon-based aqueous SC with different scan rates. Adapted with permission from Ref. [78]. **c** CV curves of carbon-based aqueous SC with various scan rates. **d** Schematic of the carbon-based SC with asymmetric redox additive electrolytes and corresponding redox reactions. Adapted with permission from Ref. [70]. **e** Schematic illustration of the self-discharge mechanism. Adapted with permission from Ref. [74]

More recently, boosting the cell voltage of aqueous SCs via introducing redox mediators has aroused considerable attentions from researchers. As an example, Li et al. established a high-voltage aqueous SCs with a potassium bromide (KBr) electrolyte [77]. Owing to the high redox potential of ${\text{Br}}^{ - } {\text{/Br}}_{{3}}^{ - }$ in neutral aqueous media, this built system was capable of delivering a high cell voltage of 1.9 V. Moreover, Hwang et al. fabricated a 2.0 V activated carbon-based aqueous ASC device with redox electrolyte [78]. Such an admirable cell voltage is ascribed to the fast redox kinetics of [Fe(CN)_6_]^4−^/[Fe(CN)_6_]^3−^ and the unique design of porous-structure electrodes (Fig. 7a, b). Similarly, Sundriyal et al. assembled a 1.8 V rGO/TiO_2_//rGO/TiO_2_ device with a redox additive electrolyte (i.e., 0.2 M K_3_[Fe(CN)_6_] in 1 M Na_2_SO_4_) [79]. It is notable that all of these examples merely added single redox mediator into aqueous electrolyte. In fact, this strategy cannot simultaneously optimize the performance of both electrodes due to the asymmetric properties of cathode and anode. For example, it was reported that the positive electrode exhibited a battery-type behavior and negative electrode displayed a pure capacitive behavior when adding hydroquinone into 1 M H_2_SO_4_ electrolyte, resulting in a big discrepancy of charge/discharge behavior of both electrodes [80]. Notably, the same phenomenon was also observed when applying other redox mediators, such as K_4_[Fe(CN)_6_], KI, and methylene blue [81–83].

In light of this, constructing a dual redox mediator electrolyte is regarded as a satisfactory strategy to remedy the above short slab. In general, dual redox mediator electrolyte is composed of two redox mediators with different redox potentials. One mediator with lower potential is applied for the negative electrode, whereas the other one with higher potential operates at the positive electrode [64]. Based on this configuration, two individual redox mediators can maximally expand the potential range of redox reactions on the two electrode surfaces, thus improving the cell voltage of intact aqueous SCs. Considering these advantages, the relevant studies of dual redox mediator electrolytes have gained substantial interests in the past years. Recently, Sun et al. exhibited a 2D carbon-based aqueous SC with dual redox mediator electrolyte [84]. Through introducing KI and anthraquinone-2-sulfonic acid sodium (AQS) into neutral KNO_3_ solution, this device with dual redox mediator electrolyte delivered a maximum cell voltage of 1.8 V and a high energy density of 33.81 Wh kg^−1^. Subsequently, Tian et al. constructed a high energy density modified activated carbon felt-based (ACF) aqueous SC with asymmetric redox additive electrolytes by adding K_3_[Fe(CN)_6_] and 2,6-dihydroxyanthraquinone (2,6-DHAQ) in 2 M KOH as positive and negative redox electrolytes, respectively [70]. Profiting from the enhancement of fast and reversible faradic reactions, this device was able to display a maximum cell voltage of 2 V and a high energy density of 39.1 Wh kg^−1^ (Fig. 7c, d).

From the foregoing, it is a favorable and efficacious avenue to increase the cell voltage of aqueous SCs through introducing redox mediator. The main advantages of this strategy are simple, cost-effective, and safe fabrication process in comparison with other methods. Furthermore, it can simultaneously enhance the capacitance and cell voltage of aqueous SCs, thereby maximizing the energy density. However, the self-discharge behavior is a main bottleneck to restrict the applications of this approach. Generally, the self-discharge phenomenon is caused by cross-diffusion and redox shuttling of charged redox couples between the oppositely polarized electrodes in the cell (Fig. 7e). This behavior may result in the low coulombic efficiency and irreversible side reactions [74]. As a consequence, it is desirable to investigate the innovative self-discharging suppression mechanisms. Besides, the number of reported redox mediators is still rare. Hence, it is a long way to find more proper redox mediators.

### Constructing “Water-in-Salt” Electrolyte

**Fig. 8:**
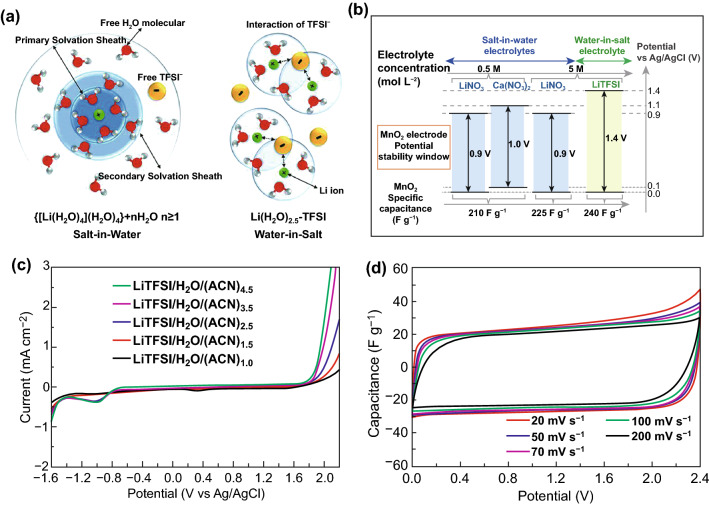
**a** Illustration of the evolution of the Li^+^ primary solvation sheath in diluted and water-in-salt solutions. Adapted with permission from Ref. [91]. **b** Potential range of MnO_2_ composite electrode in WIS and SIW electrolytes. Adapted with permission from Ref. [95]. **c** ESWs of LiTFSI/H_2_O/(ACN)_*x*_ hybrid electrolytes. **d** CV curves of the SC device using YP‐50F electrodes and LiTFSI/H_2_O/(ACN)_3.5_ electrolyte at different scan rates. Adapted with permission from Ref. [94]

It is well known that increasing the concentration of electrolyte salt is also a satisfactory and smart pathway to enlarge the cell voltage of aqueous SCs in recent years [85, 86]. In aqueous media, high concentration electrolyte salt can effectively improve the reversibility of electrochemical reactions, enhance the stability of water and reduce the yield of volatile side reaction products (e.g., CO, CO_2_, and H_2_) [87, 88]. As a typical example, “water-in-salt” (WIS) electrolyte refers to an extremely high concentration, near-saturated electrolyte, and the amount of dissociative water molecules is much lower than solute molecules [89, 90]. In 2015, Wang et al. firstly reported the definition of WIS electrolyte and applied this electrolyte to enlarge the cell voltage of aqueous lithium-ion batteries [91]. Thanks to the high solubility in water and high stability against water electrolysis, lithium bis(trifluoromethane sulfonyl)imide (LiTFSI) is selected as electrolyte salt [92]. During experimental process, 21 molality (m) LiTFSI was dissolved in 1 kg water to form the WIS electrolyte. In aqueous media, LiTFSI particles will be ionized into Li^+^ and TFSI^–^, respectively. With increase in salt concentration, the TFSI^–^ conduction band minimum and the water valence band maximum will shift to lower potentials. When the concentration of electrolyte salt attains to 21 m, this trend would lead to the decomposition of TFSI^–^ occurs preferentially on the negative electrode before HER occurs [93]. In such a super-concentrated electrolyte, almost all water molecules strongly coordinate to Li^+^, thereby suppressing the water electrolysis activity near the two electrodes (Fig. 8a) [62, 91]. On this basis, the as-synthesized highly concentrated WIS electrolyte was capable of providing a high ESW of 3.0 V and the assembled full aqueous lithium-ion battery configuration (LiMn_2_O_4_ and Mo_6_O_8_ as electrodes, respectively) demonstrated a high cell voltage of 2.3 V.

Motivated by this research, many researchers gradually pay more attention to investigating the applications of LiTFSI-based WIS electrolyte in the area of aqueous SCs [85, 94, 95]. For instance, Gambou-Bosca et al. contrasted the SC performance of MnO_2_-based electrode in the salt-in-water (SIW, 0.65 M K_2_SO_4_, 5 LiNO_3_, 0.5 M LiNO_3_, and 0.5 M Ca(NO_3_)_2_) and water-in-salt (WIS, 5 M LiTFSI) electrolytes, separately [95]. According to the experimental results, it was demonstrated that the MnO_2_ electrode displayed an extended potential range of 1.4 V (vs. SCE) and admirable specific capacitance in the WIS electrolyte which is superior to the MnO_2_ electrode operates with the SIW electrolyte (Fig. 8b). Subsequently, Zhang et al. engineered a 2.2 V hybrid aqueous ASC device which was composed of MnO_2_ (positive electrode), Fe_2_O_3_ (negative electrode), and 21 m LiTFSI (electrolyte) [21]. Similarly, a 2.2 V polyaniline-derived carbon nanorods (PDCN)-based EDLC device was also achieved by applying 21 m LiTFSI as the WIS electrolyte [24].

In spite of these great achievements, there are still two major obstacles to hinder the further development of the WIS electrolyte. One challenge is the intrinsic features of WIS electrolyte. Owing to the restriction of low conductivity and high viscosity, the WIS electrolytes with high concentration are always suffering from the under-utilization of electrode materials, resulting in the poor rate performance [62]. Aiming at this phenomenon, a co-solvent-in-salt system was reported by mixing acetonitrile with the traditional WIS electrolyte (21 m LiTFSI/H_2_O) to construct a novel “acetonitrile/water in salt” (AWIS) electrolyte [96]. According to the density functional theory-based molecular dynamics (DFT-MD) simulations, it was indicated that acetonitrile molecules could easily coordinate to lithium-ions for the remaining space of the Li^+^ solvation sheath owing to their smaller size and thus less steric interaction than TFSI^−^ anions. Hence, this AWIS electrolyte possesses an optimal concentration of 5 m, enhanced conductivity, lowered viscosity, and freezing temperature in comparison with the conventional WIS electrolyte. In view of these merits, the commercial AC-based aqueous SC device with this AWIS electrolyte could present a high cell voltage of 2.2 V. Afterwards, Xiao et al. exhibited an optimum LiTFSI/H_2_O/(ACN)_3.5_ electrolyte with the best comprehensive performance via designing a series of organic/water hybrid electrolyte systems [94]. Benefiting from the low water electrolysis activity, this electrolyte displayed an ESW as wide as 3.26 V (Fig. 8c). Besides, this resultant carbon-based symmetric SC device operating in this WIS electrolyte was able to exhibit a favorable maximum cell voltage of 2.4 V (Fig. 8d).

The other challenge is the high cost of WIS-based electrolyte. In general, the traditional WIS electrolytes are based on LiTFSI or bis(fluorosulfonyl)imide salts which are very expensive and adverse to large-scale commercial applications. To overcome this issue, it is worth searching for a series of thecost-effective soluble salts to replace the traditional LiTFSI-based electrolyte. Recently, Bu et al. reported a 17 m NaClO_4_-based WIS electrolyte to establish a high-voltage aqueous carbon-based SC system [62]. As a proper candidate, this electrolyte delivered a superior conductivity (64.2 mS cm^−1^) which was better than the traditional WIS electrolyte (8.2 mS cm^−1^). DFT-MD simulations confirmed that the majority of water molecules are coordinated with Na^+^ ions through the Lewis-basic oxygen. When an aqueous SC device was composed of the commercial AC electrodes and the NaClO_4_-based WIS electrolyte, a 2.3-V cell voltage could be achieved. In addition, an eco-friendly and cost-effective HCOOK-based WIS electrolyte was also proposed, which could reach an extremely high concentration of 40 M (mol *L*^−1^, where *L* is the volume of the mixed solution) at ambient temperature [97]. Compared to other WIS electrolytes, this electrolyte owns more negative stable potential and higher ionic conductivity. Therefore, the ESW of this electrolyte could reach 4 V (vs. Ag/AgCl) in a three-electrode system. When this electrolyte was utilized in an intact AC-based SC system, the built device could deliver a maximum cell voltage of 2.4 V.

To sum up, it is straightforward and convenient to design the high-voltage aqueous SCs via constructing the WIS electrolyte. Because of the simple operation procedure and admirable performance, this method has drawn considerable interests for the past years. However, the inferior physicochemical features (e.g., conductivity and viscosity) and exorbitant price are still impeding the further development of the WIS electrolytes. Hence, enormous efforts should be made to optimize the WIS electrolyte in the future.

## Designing Aqueous Asymmetric Supercapacitors

**Fig. 9:**
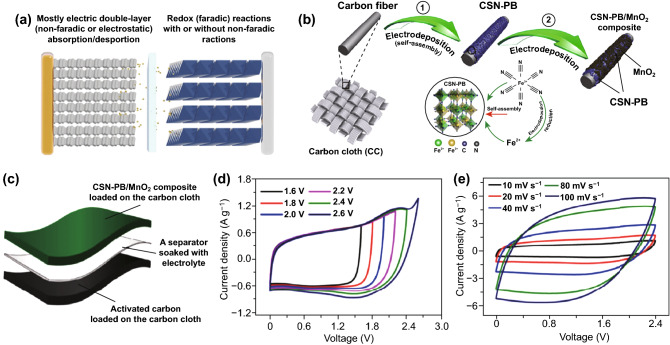
**a** Schematic of an ASC device. Adapted with permission from Ref. [50]. **b** Schematic illustration of the CSN-PB/MnO_2_//AC device configuration. **c** CV curves of the CSN-PB/MnO_2_//AC device in different potential range at a scan rate of 10 mV s^−1^. **d** CV curves of the CSN-PB/ MnO_2_//AC device at varied scan rates from 10 to 100 mV s^−1^ in a potential range of 0–2.4 V. **e** CV curves of the CSN-PB/MnO_2_//AC device at varied scan rates from 10 to 100 mV/s. Adapted with permissions from Ref. [20]

Due to the superior electrochemical performance and high economic benefit, aqueous symmetric SCs, represented by aqueous carbon-based symmetric SCs, are dominated in the commercial energy storage filed [13, 23]. Nevertheless, the intrinsic properties (e.g., low capacitance) of carbon-based electrode materials are the major drawbacks that hinder the prospect of this device. To address this challenge, asymmetric design is recognized as a promising approach to construct the high-voltage aqueous SCs [53]. Owing to the advantages of low cost and simple operation, this strategy seems to be suitable for the large-scale commercial applications. In contrast with the conventional aqueous symmetric SCs, aqueous ASC device applies a hybrid system consisted of an electric double-layer capacitive electrode and a pseudo-capacitive electrode as the energy source (Fig. 9a) [26]. Benefiting from this unique structure, aqueous ASCs normally deliver excellent electrochemical performances. During the charge and discharge process, this configuration can take full advantage of the stable potential range of two electrodes and suppress the water electrolysis process, in favor of breaking through the over-potential barrier and enlarging the cell voltage [78]. For instance, it is reported that the cell voltage of aqueous symmetric SC device is only limited to 1.2 V, whereas the ASC system can yield a maximum cell voltage as high as 2 V [53].

In view of the above merits, designing aqueous ASCs have attracted tremendous attentions to expand the cell voltage of SCs in the past years. For example, Zhang et al. assembled an advanced aqueous ASC device consisted of supramolecular network-Prussian blue (CSN-PB)/MnO_2_ composite as cathode and activated carbon as anode (Fig. 9b) [20]. Profiting from synergistic effect of the constituent components, this device can deliver an expanded cell voltage (2.4 V), high energy density (46.13 Wh kg^−1^ at 1200 W kg^−1^), and stable cyclic stability (85.5% capacitance retention after 20,000 cycles) (Fig. 9c–e). Similarly, Ghoah et al. constructed an aqueous ASC device comprised of MnO_2_/Vertical graphene nanosheets (VGN) and Fe_2_O_3_/VGN as positive and negative electrodes, respectively [98]. When this as-fabricated device operated in 1 M NaClO_4_ electrolyte, a high cell voltage of 2.6 V can be achieved. In addition, a 2.0-V wearable aqueous SC system consisted of poly(3,4-ethylenedioxythio-phene):poly(styrenesulfonic acid) (PEDOT:PSS)/Fe_2_O_3_-CNTs/CFC as negative electrode and PEDOT:PSS/CoFe_2_O_4_/CFC as positive electrode was also acquired by the asymmetric design [15].

In spite of  these great achievements, there are still two factors that restrict the prospect of high-voltage aqueous ASCs. One is the charge of two electrodes. In terms of most aqueous ASC systems, the charge of the positive and negative electrode must be balanced to ensure the cycling stability. The other is the stability of electrode materials. During the charge/discharge process, pseudo-capacitive electrode normally stores charge via redox reactions. Along with the occurrence of reactions, asymmetric device often suffers from the inferior cycling stability originating from the rapid degradation of electrode materials. Aiming at these challenges above, it is urgent to take efficacious measures to overcome these problems, such as fabricating the carbon layer coating materials and balancing the charges between two electrodes [11, 53]. Moreover, the research hot spot of aqueous SCs mainly focuses on designing high capacitance device, and the number of studies about constructing high-voltage aqueous SCs is still scarce in the last few years. Therefore, a great deal of attempts should be made to boost the cell voltage of aqueous SCs in the future.

## Conclusions and Future Perspectives

To the best of knowledge, the insufficient energy density is a major drawback of aqueous SCs, which may significantly restrict their large-scale applications. Based on the equation of *E* = 0.5*CV*^2^, it is evident that boosting the cell voltage can efficiently enhance the energy density. In this mini review, we summarize the recent advances about boosting the cell voltage of aqueous SCs, which are achieved by modifying the electrode materials, optimizing the electrolyte properties, and designing aqueous ASCs, separately. From the viewpoint of electrodes, the generic approaches include doping alkali cations, modulating the electrode mass ratio and optimizing the surface charge density, respectively. In principle, doping alkali cations into electrodes can effectively inhibit electrocatalytic activity of electrode materials and improve the over-potential of HER/OER, thereby widening of ESW of aqueous media, whereas the mechanisms of the latter two methods mainly depend on eliminating the limitation of *P*_0V_ and improving the utilization efficiency of electrode potential range. From the viewpoint of electrolytes, the universal pathways contain adjusting the appropriate pH level, introducing redox mediators and constructing “water-in-salt” electrolyte, respectively. The mechanism of aforesaid pathways is suppressing the water electrolysis activity. In addition, the mechanism of designing ASCs is enhancing the potential range utilization of both electrodes. Although great achievements have been obtained for constructing high-voltage aqueous SCs in recent years, there are still several bottlenecks to break through in the subsequent working process. On the basis of relevant studies, we summarize the as-following challenges and future development trends:

1. The diversity of doping particles. Except for the alkali cations, doping other categories particles (e.g., alkaline earth cations, halide ions, and acid group cations) into electrode materials is also worthwhile exploring in the future.
2. Simplifying the operation procedures of modification pathways. Taking modulating the electrode mass ratio for an example, due to the lack of definite theoretical analysis methods, it is time-consuming and complicated to obtain the optimal electrode mass ratio through various experimental gradient designs. Therefore, it is urgent to take smart and accurate measures to simplify the experimental process.
3. Eliminating the adverse influences of self-discharge behavior. In the aforesaid sections, we have discussed that applying ECI technique and constructing redox electrolytes normally suffer from the self-discharge effect, leading to the poor electrochemical ability. As a consequence, how to suppress the self-discharge behavior is still a challenging research in the subsequent process.
4. Optimizing the features of WIS electrolyte. Although constructing WIS electrolyte is more efficient and straightforward to widen the cell voltage of aqueous SCs, the intrinsic shortcomings (e g., low conductivity, high viscosity, and expensive price) of traditional LiTFSI salt are the major drawbacks to hinder its commercial applications. Hence, it is desirable to optimize the traditional LiTFSI-based electrolytes and search for the cheap substitutes for LiTFSI salt.
5. Combining the merits of modifying electrodes and electrolytes. In common, adopting single strategy from electrode or electrolyte aspect cannot satisfy the demands of high-voltage aqueous SCs. Consequently, it is worth simultaneously optimizing the properties of electrodes and electrolytes to boost the cell voltage of aqueous SCs.
6. Improving the cycling stability of electrode materials. For the most aqueous ASC devices, the electrode materials often suffer from the stability issue during the repeated cyclic process, thereby causing the inferior electrochemical performance. Hence, some efficacious measures should be taken to solve this problem, such as designing nano-structure electrodes and introducing carbon-based layer coating materials.

In summary, the purpose of this mini review is expected to attract more attentions to the relevant studies about constructing high-voltage aqueous SCs and provide referencing values for other researchers, thereby promoting the further progress of aqueous SCs with high energy density.
